# The Promise of Human Induced Pluripotent Stem Cells in Dental Research

**DOI:** 10.1155/2012/423868

**Published:** 2012-05-09

**Authors:** Thekkeparambil Chandrabose Srijaya, Padmaja Jayaprasad Pradeep, Rosnah Binti Zain, Sabri Musa, Noor Hayaty Abu Kasim, Vijayendran Govindasamy

**Affiliations:** ^1^Research and Development Department, Hygieia Innovation Sdn. Bhd, Lot 1G-2G, Komplex Lanai, No.2, Persiaran Seri Perdana, Persint 10, Territory of Putrajaya, 62250 Federal, Malaysia; ^2^Department of Conservative Dentistry, Faculty of Dentistry, University of Malaya, 50603 Kuala Lumpur, Malaysia; ^3^Oral Cancer Research and Coordinating Centre (OCRCC), Faculty of Dentistry, University of Malaya, 50603 Kuala Lumpur, Malaysia; ^4^Department of Oral Pathology, Oral Medicine and Periodontology, Faculty of Dentistry, University of Malaya, 50603 Kuala Lumpur, Malaysia; ^5^Department of Children's Dentistry and Orthodontics, Faculty of Dentistry, University of Malaya, 50603 Kuala Lumpur, Malaysia

## Abstract

Induced pluripotent stem cell-based therapy for treating genetic disorders has become an interesting field of research in recent years. However, there is a paucity of information regarding the applicability of induced pluripotent stem cells in dental research. Recent advances in the use of induced pluripotent stem cells have the potential for developing disease-specific iPSC lines *in vitro* from patients. Indeed, this has provided a perfect cell source for disease modeling and a better understanding of genetic aberrations, pathogenicity, and drug screening. In this paper, we will summarize the recent progress of the disease-specific iPSC development for various human diseases and try to evaluate the possibility of application of iPS technology in dentistry, including its capacity for reprogramming some genetic orodental diseases. In addition to the easy availability and suitability of dental stem cells, the approach of generating patient-specific pluripotent stem cells will undoubtedly benefit patients suffering from orodental disorders.

## 1. Introduction

Human embryonic stem cells (hESCs) are pluripotent cells, which have remarkable proliferation ability to differentiate into any cell types of all three germ layers in a defined culture condition. Hence embryonic stem cells have been regarded as the most potent tool for experimental studies, drug screening, and regenerative medicine [1]. However, the ethical dilemmas regarding the donation or destruction of human embryos and the immunoincompatibility of hESCs have impeded its application in cell-based therapy [1]. In order to overcome these problems, reprogramming techniques have been introduced where somatic cells can be reversed into a pluripotent stem cell-like state. It is generally believed that induced pluripotent stem (iPSC) cells might demonstrate the potential for alleviating incurable diseases and aiding organ transplantation [2]. 

It has been shown that iPSCs can be derived efficiently from various human cell types [3–8]. An interesting observation is that the transcriptional and epigenetic features of iPSCs are reported to be similar to hESCs [9–11]. Nevertheless, further insights into the inherent similarities and differences between hESCs and iPSCs would be advantageous in understanding the reasons why the use of hESCs in clinical and translational applications has been held back [12, 13]. 

## 2. Generation of Induced Pluripotent Stem Cells

Induced pluripotent stem cells can be produced by forced expression of certain genes by reversing them to a pluripotent state similar to that of embryonic stem cells (ESCs). However, the generation of iPSC requires extremely safe and efficient approaches or strategies to decrease the risk of tumors that may result from the introduction of undifferentiated iPSCs into patients. Though such constraints prevail, the approach of generating patient-specific pluripotent cells will undoubtedly benefit regenerative medicine in many ways [14]. The first iPSCs generation was reported by Takahashi and Yamanaka [15] in 2006. They generated the iPSCs through simultaneous overexpression of a group of transcription factors using cell lines derived from mice. A similar genetic manipulation approach was used to generate pluripotent stem cells from human fibroblast cells [11, 13, 16]. In addition to this approach, other modes have also been devised for iPSCs generation including; single polycistronic vector [17], nonintegrating adenoviral APS approaches [18, 19], the PiggyBac transposon system, which removes the transgenes from established iPSC lines after inducing pluripotency [20, 21], the Cre/lox Precombination system [22], and nonintegrating “episomal” vectors that create iPSCs free of vector and transgene DNA [23]. As these methodologies depended solely on foreign DNA transfer into target cells, protein-based methods have been introduced to address safety issues. In these methods various reprogramming proteins are delivered into cells by conjugating them with a short peptide that mediates protein transduction, such as HIV tat and polyarginine [24, 25]. In addition, an alternative approach has been described which uses synthetic mRNA to induce pluripotency and differentiation [26]. This new approach showed superior conversion efficiency and kinetics than the earlier described protocols. This mode of cellular reprogramming is a holistic approach as this transfers all the regulatory components from a target cell to a donor cell. Moreover, the cellular reprogramming is achieved by manipulating the whole genome system rather than a small set of master genes. 

Therefore finding a safe and efficient mode of iPSCs generation requires a better understanding of the biology of cellular reprogramming. Even though live cells are the phenotypic representations of their genomic state (gene-regulation, epigenetic modifications, and cellular physiology), they do not have a steady molecular state [27]. For this reason, it is possible for the cells to be switched or reprogrammed into a pluripotent state, even in their differentiated form. 

## 3. Characterization of iPSC Lines

Generation of iPSC lines were always followed up by subsequent characterizations to ensure the purity and quality of the generated cells and their pluripotency potential. One of the most convenient and direct methods developed for detecting and isolating iPSC was by live immunocytochemistry [28]. Using this technique the characterization of pluripotency can be achieved using intracellular and cell-surface biomarkers such as SSEA-3, SSEA-4, Tra-1-60, and Tra-1-81 [29]. In addition, flow cytometry analyses helps to quantify the expression of these markers at the individual cell level. 

In addition to live staining, auxiliary identification was demonstrated using alkaline phosphatase (AP) staining for the reprogramming factors, as AP is a universal marker in the identification of iPSCs [29]. Further evaluation of pluripotency is performed through semiquantitative and quantitative polymerase chain reactions (PCRs) through the expression of both endogenous genes and transgenes [30]. This is followed up by the analysis of methylation status of the promoter region of pluripotent genes by bisulfite sequencing of the CpG islands [29]. Karyotyping analysis is also carried out using standard G-banding chromosome analysis to determine chromosome stability of iPS cell lines [29]. Further, *in vitro* differentiation of pluripotent stem cells is characterized by the formation of embryoid body followed by teratoma assays [30]. This assay is used to confirm formation of all three embryonic germ layers [30]. 

## 4. Advances in Disease-Specific iPSCs and Their Applications

Although most of the human-related disease studies are undertaken using rodent models, a genetic defect or disorder produced in human does not necessarily cause the same symptoms in rodents. Therefore, cell cultures from human tissues are considered to be the most suitable complement to animal models. The iPSC technology has made the production of disease-specific stem cells that carry the genome of the donor possible and it mimics the human diseases more reliably than animal models. Apart from generating an *in vitro* model, disease-specific iPS cell lines from different individuals also allow better understanding of the nature and complexity of a disease. At present the most immediate requirement of such a human disease model is to explore the progression of a disease in different tissues of the human body and also to compare the variability among patients [2]. 

## 5. Existing Types of Disease-Specific iPSC Lines

A number of studies have been conducted on disease-specific iPSC lines and some of them have provided understanding of the disease mechanisms.  Table 1 summarizes the up-to-date literature in which human disease-specific iPSC lines have been generated. The most convincing fact for commencing these studies using iPSC technology was that disease-specific pluripotent cell lines could be generated successfully from patients with a variety of genetic disorders where the iPSC lines had similar characteristic capacity, equivalent to those from a normal individual [11]. Moreover, these iPSC lines were able to differentiate into required cell types of relevant diseases and recapitulate disease-specific effects *in vitro* which may not be detectable in animal models [31, 32].

## 6. Perspective of iPS Technology in Dental Research

Initially the concept of utilizing iPSCs technology to model disease was mostly emphasized in neural degenerative diseases, which was then extended to other genetic disorders including immune system, muscular, blood, pancreas, skin, bone marrow, liver, lung, retinal, premature ageing, as well as other physical and intellectual disorders. However, the concept of utilizing iPSCs technology is still in its infancy for orodental disorders and diseases. Chronic degenerative dental diseases are widespread in human populations and represent a significant problem for public health. The iPS technology and its application in treating orodental diseases could be a powerful therapeutic tool in dentistry.

Most of the diseases and disorders have a major genetic component. Human diseases and disorders may result from single-gene mutations, but more commonly they are complex as a consequence of multiple gene-gene or gene-environment interactions [60]. The cause of the majority of orodental diseases could be genetically related if infection and traumatic effects are not taken into account. The characteristic signs and symptoms of these diseases indicate genetic origin [61–63], although not all have been clearly identified.

Globally, every year an average of 7% of infants have some mental or physical defect. Among these, 75% are related to craniofacial defects or malformations [64]. Again it is the dental anomalies that form an integral aspect of such genetic disorders, often representing important clinical clues to the true underlying disorders. Specific examples that are well documented include (1) ectodermal dysplasia [65] with dental manifestations of oligodontia and conical shaped teeth and (2) cleidocranial dysplasia with multiple supernumerary and unerupted teeth [66, 67]. Therefore, it is necessary for dentists to be aware of the clinical characteristics and the possible alterations that are part of the genetic syndromes, so that they can offer patients multidisciplinary and the best possible treatments. Some of the documented examples of these types of direct or indirect genetic alterations causing dental defects are listed in Table 2.

Possibly, iPSC possess the potential for treating such genetic orodental disorders, confining the availability of suitable disease-specific iPSCs from the diseased person which are able to multiply, cooperate and reform the missing or diseased part. Though, multiple types of stem/progenitor cells have been identified based on their ability to repair/regenerate and partially restore organ function in the human body, growing evidence illustrates that stem cells are primarily found in niches and that certain tissues contain more stem cells than others [94].

## 7. Dental Stem Cell Niches as a Potential Source for Human iPSCs Generation

The foundation of personalized medicine profoundly lies on procuring the most suitable cell sources. In the human body, various cell sources have been shown to be reprogrammed into iPSC. Among these are dermal fibroblasts, the first of the cell types to be reprogrammed into iPSC, followed by other sources like amniotic fluid-derived cells, skin keratinocytes, embryonic stem cell-derived fibroblasts (ESFs), CD34 blood cells, mesenchymal stem cells (MSCs), and dental pulp [7]. However, studies are showing that it is easier to reprogram more immature cells than somatic cells. Hence immense research was carried out to refine the methodology of iPS technology in terms of techniques, efficiency, and cell type choice. It has been reported that reprogramming efficiency for human fibroblasts is relatively low, while the reprogramming process for keratinocytes generates iPSC colonies 100-fold more efficiently and 2-fold faster as compared to human fibroblasts [95]. The probable cause for such efficacy difference is that keratinocytes have expression levels of stem cell-related genes more similar to ESC than fibroblasts [95]. 

A similar comparable study reported that dental tissue-derived mesenchymal-like stem cells can be reprogrammed into iPSCs more efficiently, when compared to other mature somatic cells from human body such as neonatal foreskin fibroblasts, adult MSCs, and adult dermal fibroblasts [7]. This is probably because of the timing and other factors required for reprogramming a somatic cell to iPS varies greatly depending on cellular context. For example, the reprogramming of MSCs from somatic cell sources mentioned above requires the addition of hTERT (telomerase reverse transferase) and SV40 large-T to turn into iPSCs, whereas dental tissue-derived cells are not confined the same way [7]. Perhaps this emphasizes the use of dental pulp as the most feasible and rich source of mesenchymal stem cells to be used in regenerative therapy, as they are easily available when compared to the tedious collection procedure of other somatic cells. 

**Figure 1:**
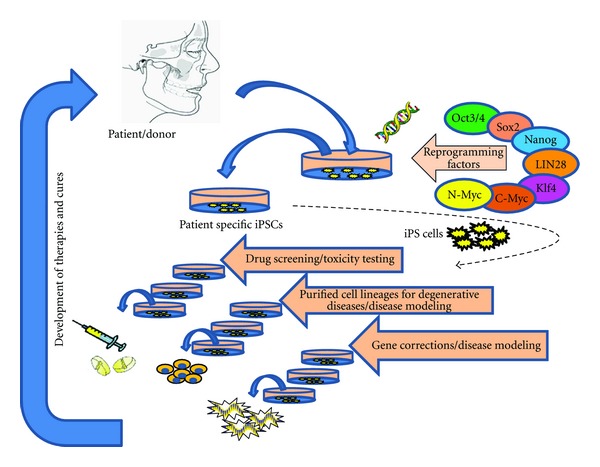
A schematic representation of the human iPSCs generation.

Dental stem cells can be easily obtained from the pulp of exfoliated primary teeth (SHED) or extracted primary (SCD) and permanent (DPSC), apical papilla (SCAP), tooth germs, and human periodontal ligament. In fact, all these cells can be successfully reprogrammed into iPS cells [94]. A recent report further strengthens the potential of dental-derived stem cells, where reprogramming of human immature dental pulp stem cells (hIDPSCs) was successful within a short-time frame as compared to human fibroblasts, SHED, and DPSC. Furthermore, primary hIDPSC-iPSC colonies were readily obtained even under feeder-free conditions eliminating the possibility of contamination from xenoenvironment [95]. The physiologically intact dental pulp stem cells could be successfully differentiated to advanced derivatives of all three primary germ layers (odontoblast, osteoblast, chondrocyte, myocyte, neurocyte, adipocyte, corneal epithelial cell, melanoma cell, iPSC) (refer review, [94]). Collectively, its multipotency, high proliferation rates, and accessibility make the dental stem cell an attractive source of mesenchymal stem cells for iPS generation. Hence dental-derived stem cells should be considered as a strategy in future regenerative therapies. A schematic representation of the human iPSCs generation from dental stem cells and its applications in various therapeutic approaches is shown in Figure 1.

## 8. Therapeutic Potentials of Disease-Specific iPSCs for Genetic Orodental Diseases/Disorders

Mutations have been shown to play a dominant part in most orodental diseases as tabulated in Table 2 and these genetically caused diseases are the ones that could benefit the most from iPS technology. One of the main focuses of the present stem cell therapy is genetic correction, which would be a permanent solution. For example the iPSCs has shown its therapeutic capability to treat diseases by correcting the underlying genetic defects, which was successfully demonstrated in mouse models of sickle cell anemia [96]. The defective gene was replaced by wild type *β*-globin by homologous recombination. Surprisingly, the genetically corrected iPSC-derived hematopoietic progenitor was effective in improving and restoring the physiological function of the diseased animal. This proof of principle was also introduced in human individuals with Fanconi anemia, a disease characterized by severe genetic instability [54]. Hence, this approach can be applied to any genetic disease underlying the human body. Recent studies have also shown the possibilities of developing human endoderm tissue-derived iPSC lines. This, along with other established human iPSCs lines, has provided a base to elucidate the mechanisms of cellular reprogramming and also to study the safety as well as efficiency of differentially originated human iPSCs [27]. Studies on liver pathogenesis using iPSCs technology have provided a more amenable system to generate liver disease-specific cell lines. The ability to develop such disease-specific stem cell models can be utilized for disease modeling which helps in the study of the complicated pathogenesis and drug screening purposes [27]. Similarly, studies have also been undertaken for neural degenerative diseases like Parkinson's disease and retinal disease (Retinitis pigmentosa; gyrate atrophy) (Table 1).

Most clinical therapies and treatments on disorders of neural, retinal, hepatic, diabetic, bone, and tissue aberrations are mostly focused on only particular tissue aspects of human body. However, some of these disorders have orodental manifestations. Moreover, most of the identified genetic orodental diseases are also encountered with similar problems as those of other disorders. In this context, the use of iPSC therapy for treating such disorders that were applied earlier can also be considered for orodental diseases. The promise of regenerative medicine in orodental disease is reinforced with the potential applicability of stem cell therapy in dentistry, which could provide an ideal solution to certain prevailing problems. For example, an immature tooth with extensive coronal and pulp damage could be reversible through regeneration of tooth tissues. Similarly, regeneration of resorbed root, cervical, or apical dentin, periodontal regeneration, whole-tooth regeneration, repair and replacement of bone in craniofacial defects can facilitate restoring the physiologic structural integrity [97]. For instance, the successful regeneration of periodontal tissue, alveolar bone, cementum, and periodontal ligament has been achieved using autologous periodontal ligament mesenchymal stem cells (PDL-MSCs), with no adverse effect when transplanted [98]. Considering the success of such attempts using tissue engineering techniques, by applying the advanced iPS cell technology, more fruitful advantage can be expected for their use in cell transplantation therapies and gene corrections in orodental disorders. 

The regeneration of orodental tissues is dependent on four basic components. The appropriate signals, cells, blood supply, and scaffold that are needed to target the tissue at the site of defect [99]. These four elements play a fundamental role in the reconstruction and healing of lost tissues. The cells provide the machinery for new tissue growth and differentiation, whereas the growth factors modulate the cellular activity and stimulate the cells to differentiate as well as produce tissue matrix [99]. The new vascular tissues provide the nutritional base for tissue growth and the scaffolds guide and create a template structure in three-dimensions to facilitate the tissue regeneration process [99]. Tissue engineering strategies using this basic cell transplantation approach can be successfully applied for a wide variety of oral structures such as bone, periodontal ligament, oral mucosa, skin, and teeth. In addition, such cells can also be genetically modified *ex vivo* by using iPS technology and thereby merging stem cell technology and precision gene therapy, a new therapeutic approach for oral genetic disorders is possible. This impulses the possibility of their use in iPS technology, since they can be utilized not only for dental associated problems, but may facilitate the repair of nondental tissues such as bones and nerves [61, 100, 101]. 

Hence, if we could attempt the real possibility of *ex vivo* genetic manipulations, iPSCs will be the most powerful therapeutic tools for a variety of dental pathologies which have yet to be investigated. In this regard, it is valuable to establish disease-specific iPSC lines, preferably for the genetic dental disorders to comprehensively evaluate their disease modeling potentials. Therefore fundamental research program is needed to ascertain the application of iPS technology in genetic orodental disorders, which requires extensive programs that can be directed to each aspect of dental diseases and its genetic cause. 

## 9. Conclusion

Though studies have reported the successful generation of disease-specific iPSC lines from individuals with different diseases, effective disease modeling has been demonstrated only by a few studies. The development of iPSC models for orodental diseases is still a new concept. The availability of such iPSC models will lead to better understanding of the nature and behavior of orodental diseases. Possibly the opportunities for the exploration of iPS technology in treating orodental diseases will lead to a significant benefit for the population at large.

## Figures and Tables

**Table 1:** Disease-specific-induced pluripotent stem cells (iPSCs) lines from various human genetic disorders.

Disease category	Disease	References
Neural	Amyotrophic lateral sclerosis	[31]
	Parkinson's disease	[11, 22, 33, 34]
	Huntington's disease	[11, 35]
	Lesch-Nyhan syndrome	[11]
	Rett syndrome	[36]
	Familial dysautonomia	[37]
	Angelman syndrome	[38]
	Prader-Willi syndrome	[38, 39]
	Friedreich's ataxia	[40]
	Rett syndrome	[41]
	Schizophrenia	[42]

Immune system	ADA-SCID	[11]
	Scleroderma	[43]
	Primary immunodeficiency	[44]

Muscular	Duchenne muscular dystrophy	[11]
	Becker muscular dystrophy	[11]
	Spinal muscular atrophy	[25]
	Duchenne muscular dystrophy	[45]

Blood	Thalassemia	[46, 47]
	Sickle cell anemia	[43, 47]
	Chronic myeloid leukemia	[48]

Heart	Long QT syndrome	[49, 50]

Pancreas	Juvenile diabetes mellitus	[11]
	Shwachman-Bodian-Diamond syndrome	[11]
	Type I diabetes	[51]

Skin	Leopard syndrome	[52]
	Recessive dystrophic, Epidermolysis bullosa	[53]

Bone marrow	Fanconi anemia	[54]
	Myeloproliferative diseases	[54]

Liver	Liver diseases: a1-antitrypsin deficiency, familial hypercholesterolemia, glycogen storagedisease type 1a, Crigler-Najjar, tyrosinemia type 1	[55]

Lung	Lung diseases: cystic fibrosis, a-1 antitrypsin deficiency-related emphysema	[43]

Others (physical and intellectual limitations)	Down syndrome	[11]
	Hurler syndrome	[44]
	Gaucher disease	[11]
	Fragile X syndrome	[56]

Premature ageing	Dyskeratosis congenital	[57]

Eye	Retinitis pigmentosa	[58]
	gyrate atrophy	[59]

Dental	?	

**Table 2:** Human genetic oral diseases/disorders causing dental defects.

Dental disease/disorder	Symptoms	Genetic cause	References
Orofaciodigital syndrome 1 (OFD1)	Malformations of the face, oral cavity, oral clefts, underdeveloped nose flaps, finger abnormalities, hydronephrosis, and variable involvement of the central nervous system.	Mutations in OFD1 gene; mutations in the Cxorf5 gene, located in the Xp22	[68–70]

Oculofaciocardiodental (OFCD)	Canine radiculomegaly; oligodontia, delayed eruption of the dentition, malocclusion, root dilacerations, macrodontia, and enamel defects; microphthalmia and, congenital cataracts with secondary glaucoma	Mutations in the BCOR gene located in the chromosome Xp11.4	[71, 72]

Amelogenesis imperfecta (AI)	Developmental abnormalities in the quantity and/or quality of tooth enamel, occasionally in conjunction with other dental, oral, and extraoral tissues	Mutations in any of the six genes AMELX, ENAM, MMP20, KLK4, FAM83H, and WDR72	[73]

Cherubism	Bilateral bone enlargement of the jaws in childhood; displacement or aplasia of teeth and tooth-germs	Mutations in the gene encoding the binding protein SH3BP2 on chromosome 4p16.3	[74]

Disorders of human dentin: (a) dentinogenesis imperfectas (DI, types I–III) (b) dentin dysplasias (DD, types I and II)	Discoloured teeth (brown-blue or opalescent brown) and structural defects such as bulbous crowns and small pulp chambers	Mutation in dentin sialophosphoprotein gene (DSPP, 4q21.3)	[75]

Periodontal disease	Inflammatory as well as recessive alterations of the gingiva and periodontium	Mutation in interleukin-1 (IL-1) gene	[76, 77]

Hypodontia	Missing one to six teeth (excluding the third molars)	Mutations in transcriptions factors of MSX1 gene in chromosome 4 or another transcription factor gene PAX9 in chromosome 14	[78, 79]

Cleidocranial dysplasia (CCD)	Affects the bones of the face causing a wide skull, a prominent forehead, a flat nose and a small upper jaw; delayed resorption and shedding of primary teeth, delayed maturation, and partial or absent eruption of the permanent teeth combined with ectopic position and development of cysts around the nonerupted molar	Mutation in the RUNX2 (CBFA1) gene found on chromosome six, 6p21.1	[80–82]

Some dermatological syndrome causing oral and dental manifestation			

Congenital erythropoietic porphyria	Hemolytic anemia, photosensitivity (manifested as blistering of the skin), skin fragility, mutilating scarring, hypertrichosis and hyperpigmentation, and deposition of red-brown pigment in the bones and teeth; oral mucosa is pale and the teeth have a red to maroon color	Mutations in the UROS gene which is located in the locus 10q25.2–q26.3	[83]

Ectodermal dysplasias	Characterized by the observation of anodontia and hypodontia of the temporal and permanent dentition, impacted teeth, pin-type dental malformations, enamel hypoplasia, multiples diastemas, and underdeveloped alveolar ridges	Mutation of Xq12–q13.1 (XLHED-gene) and also mutations in the *TP63 *gene	[84, 85]

Epidermolysis bullosa	Repeated blistering, the formation of scars, limitation of oral aperture, ankyloglossia, disappearance of the oral and vestibular sulci, perioral stenosis, severe periodontal disease and bone reabsorption, atrophy of the upper maxilla with mandibular prognathism, an increased mandibular angle, and a predisposition to oral carcinoma	Mutations in either the keratin 5 (KRT5) or keratin 14 (KRT14) gene	[86, 87]

Gardner syndrome	Epidermoid cysts, desmoid tumors, and other benign tumors; supernumerary teeth, compound odontomas, hypodontia, abnormal tooth morphology, and impacted or unerupted teeth	Mutation in the APC gene located in chromosome 5q21. High-resolution banding analysis showed an interstitial deletion of the long arm of chromosome 5 (q22.1→q31.1)	[88]

Incontinentia pigmenti	Distinctive swirling pattern of the skin; defects of teeth, hair, and nails; ophthalmic, central nervous system, and musculoskeletal abnormalities	Mutations in the NEMO gene that completely abolishes expression of NF-kappaB essential modulator	[89]

Naegeli-Franceschetti-Jadassohn syndrome	Affects the sweat glands, skin, nails, and teeth; reticulated hyperpigmentation, hypohidrosis, palmoplantar hyperkeratosis, abnormal teeth, and nail dysplasia; abnormally shaped teeth, polydontia, yellow spotted enamel, caries, and early total loss	Mutations in the keratin 14 (KRT14) gene, located on chromosome 17q11.2–q21	[90]

Papillon-Lefevre syndrome	Palmoplantar hyperkeratosis and rapid periodontal destruction	Mutations of a gene that regulates production of an enzyme known as cathepsin C, located on the long arm (q) of chromosome 11 (11q14–q21)	[91]

Sjogren-Larsson syndrome	Congenital ichthyosis, spastic diplegia or quadriplegia, and mental retardation; white dots in the fundus, speech defects, epilepsy, dental problems, and skeletal abnormalities	Mutations in the FALDH (ALDH3A2) gene on chromosome 17p11.2	[92, 93]
